# 2D Magnetic Materials for Sensor Technologies

**DOI:** 10.3390/s26082467

**Published:** 2026-04-17

**Authors:** Matthew Metcalf, Bamidele Onipede, Jesse Martinez, Hui Cai

**Affiliations:** 1 Department of Physics, University of California, Merced, Merced, CA 95343, USA; mmetcalf@ucmerced.edu (M.M.); bonipede@ucmerced.edu (B.O.); jmartinez564@ucmerced.edu (J.M.); 2 Materials Sciences Division, Lawrence Berkeley National Laboratory, Berkeley, CA 94720, USA

**Keywords:** two-dimensional magnetic materials, van der Waals magnets, magnetic sensing, spintronics

## Abstract

Two-dimensional (2D) magnetic materials have emerged as a promising platform for next-generation sensing technologies due to their atomic thickness, tunable magnetic properties, and compatibility with van der Waals heterostructures. Rapid progress in material discovery, synthesis, and device integration has expanded opportunities for compact, low-power, and highly sensitive sensor platforms. This review examines selected sensing mechanisms enabled by 2D magnetic materials, highlighting recent experimental advances and emerging device concepts. Current limitations and challenges such as environmental stability, scalability, and room-temperature operation are considered in the context of ongoing research efforts. By examining these approaches, this review aims to provide insight into the current development and potential of 2D magnetic materials for sensing technologies. This review is organized to first introduce the fundamental properties and challenges of 2D magnetic materials, followed by a survey of key sensing mechanisms and representative device implementations, and concludes with an outlook on future research directions.

## 1. Introduction

Sensors are central to modern technologies, including navigation systems, robotics, data-storage readout, current and position sensing, biomedical instrumentation, wearable electronics, and adaptive electronic platforms [1,2,3,4]. These applications require miniaturized, energy-efficient, and ultrasensitive devices that can overcome the limitations of traditional three-dimensional semiconductors and bulk materials, motivating growing interest in alternative materials for next-generation sensing platforms [5,6,7,8]. Among the candidate material platforms, two-dimensional (2D) materials have attracted particular attention as a result of their atomic thickness, strong electrostatic tunability, high surface sensitivity, and simple integration into van der Waals (vdW) heterostructures which enable compact and multifunctional sensor architectures [9,10]. Recent studies have further emphasized that, beyond material discovery, advances in fabrication strategies and interface control within 2D heterostructures are critical for enabling scalable and reliable device integration [11].

Formulated in 1966, the Mermin–Wagner theorem states that long-range magnetic order cannot exist in isotropic two-dimensional Heisenberg systems at finite temperature, owing to the destabilizing effect of thermally excited spin wave fluctuations [12]. This theorem for a long time discouraged the experimental search for intrinsic 2D magnets, and early investigations of magnetic thin films primarily emphasized proximity-induced, interfacial, or defect-mediated magnetism rather than true monolayer magnetic order. However, in 2004, graphene was successfully isolated [13], demonstrating that single-atom-thick crystals could be isolated, characterized, and integrated into a device while exhibiting properties different from the bulk. This led to a fundamental shift centered around the question as to the stability of long-range magnetic order in anisotropic 2D materials. The first experimental evidence emerged in 2016, when Lee et al. demonstrated Ising-type antiferromagnetic order persistent down to the monolayer limit in FePS_3_ [14], a layered transition metal whose strong uniaxial anisotropy circumvents the Mermin–Wagner constraint and stabilizes the ordered phase with a Néel temperature of approximately 118 K independent of thickness. In 2017, two simultaneous reports confirmed the presence of long-range ferromagnetic order in atomically thin vdW crystals: Gong et al. observed ferromagnetism in few-layer Cr_2_Ge_2_Te_6_ via magneto-optical Kerr effect microscopy [15], and Huang et al. demonstrated layer-dependent ferromagnetism in monolayer CrI_3_, with the monolayer exhibiting out-of-plane Ising ferromagnetism while the bilayer adopted a layered antiferromagnetic ground state [16]. These discoveries triggered a period of rapid expansion which saw giant tunneling magnetoresistance reported in vdW magnetic tunnel junctions based on CrI_3_ [17,18,19] and electrostatic gating switching of the interlayer magnetic order in bilayer CrI_3_ [20]. In the same year, a critical milestone for ambient device applications was made when ionic gating of few-layer Fe_3_GeTe_2_ was found to elevate its ferromagnetic transition temperature to room temperature [21]. This rapid progression from a theoretically forbidden phenomenon to a rich platform for device engineering within little more than a decade established 2D magnetic materials as one of the most dynamically evolving frontiers in condensed matter physics and materials science and set the stage for their systematic exploration as active elements in next-generation sensor technologies.

2D magnetic materials offer transformative physical and structural attributes uniquely suited for advanced magnetic sensor architectures. With thicknesses typically ranging from 0.7 to 1 nm, 2D magnets bypass the detrimental “size effects” that severely degrade the magnetic performance of bulk materials at the nanoscale [22]. This atomic-scale thinness not only permits the ultimate miniaturization of components, but also drives massive increases in the signal-to-noise ratio [23,24,25]. Furthermore, because magnetic field strength decays rapidly with distance, achieving high sensitivity requires minimizing the gap between the sensor and the source [26]. In 2D materials, the entire active volume is effectively on the surface. This unique geometry allows magnetic probes to be placed in unprecedented proximity to target sources, enabling highly localized nanoscale magnetometry. Ultimately, these attributes translate directly into enhanced transduction efficiency, allowing 2D-based devices to detect significantly weaker magnetic stimuli with a thinner active layer and a vastly smaller footprint than conventional sensor architectures can achieve [27]. Beyond geometric advantages, the exceptional electrostatic tunability of 2D magnets is another advantage. Because carrier densities in atomically thin layers can be modulated continuously and reversibly by a proximal gate electrode without the screening limitations of bulk systems, both the magnitude and the character of the magnetic order, including the Curie or Néel temperature, the coercive field, and even the sign of the interlayer exchange coupling are directly accessible as control parameters [20,28,29]. The van der Waals nature of 2D materials provides another advantage that is particularly important for device integration. As adjacent layers are held together by weak dispersion forces rather than covalent or ionic bonds, arbitrary combinations of magnetic, semiconducting, insulating, superconducting, and topological 2D materials can be assembled into heterostructures with atomically clean, lattice-mismatch-free interfaces [30,31]. In addition to traditional van der Waals materials that are commonly employed in these heterostructures, other 2D and quasi-2D materials such as Xenes/transgraphenes [32,33] (graphene-like mono-elemental 2D materials; e.g., silicene, germanene) offer tunable electronic and spin-dependent properties that may serve as functional components in engineered stacks. Finally, the mechanical compliance of atomically thin membranes which can sustain strains of several percent without fracture opens pathways to flexible, wearable, and bio-interfaced sensor platforms that are incompatible with rigid bulk materials [15].

While offering immense promise, designing reliable platforms with 2D magnetic materials requires navigating several intricate physical and practical complexities. In the strict 2D limit, long-range magnetic order is destabilized by thermal spin fluctuations, as described by the Mermin–Wagner theorem. This limitation is overcome in real materials by relying heavily on magnetic anisotropy to create an energy gap in the spin-wave spectrum that prevents the excitation of long-wavelength magnons, thereby stabilizing the long-range magnetic order at finite temperatures [34,35]. The resulting ordered state depends on spin dimensionality and is conventionally described within three statistical frameworks. In the Ising model, discrete out-of-plane spins can support true long-range order at finite temperature. In the XY model, in-plane spins exhibit quasi-long-range order with algebraically decaying correlations and undergo a topological Berezinskii–Kosterlitz–Thouless transition rather than a conventional symmetry breaking transition [36,37]. In contrast, the isotropic Heisenberg model does not sustain true long-range order in the ideal 2D limit without external or intrinsic symmetry breaking [12]. Beyond determining the stability of long-range order, the microscopic origin of magnetic anisotropy introduces additional complexity. Spin–orbit coupling, crystal-field symmetry, superexchange geometry, Kitaev interactions, and the Dzyaloshinskii–Moriya interaction (DMI) collectively shape the magnetic ground state [38,39]. In systems lacking inversion symmetry, the DMI competes with isotropic exchange to select among collinear, canted, spiral, and topologically non-trivial spin textures [39]. This competition can stabilize skyrmions in heterostructures where interfacial symmetry breaking is enhanced [40]. While skyrmions offer opportunities for topological Hall sensing, their stochastic nucleation and annihilation introduce unpredictable noise in devices not explicitly engineered to exploit them. These intrinsic complexities are compounded by a pronounced sensitivity to layer number and stacking configuration. Magnetism in 2D systems is governed by competing mechanisms: direct exchange, ligand-mediated superexchange, double exchange, and interlayer Ruderman-Kittel-Kasuya-Yosida (RKKY) coupling in metallic systems leading to macroscopic behavior shifting rapidly with thickness [34]. For example, in CrI_3_, the monolayer is an Ising ferromagnet, the bilayer is antiferromagnetic due to monoclinic stacking driven superexchange, and the trilayer reverts to ferromagnetic order [16]. Small, uncontrolled variations in flake thickness therefore produce qualitatively different magnetic responses in nominally identical devices. Rotational misalignment between layers introduces yet another degree of freedom through moiré magnetism. The resulting superlattice modulates interlayer exchange, stabilizing coexisting ferromagnetic and antiferromagnetic domains or ordered arrays of topological spin textures that depend sensitively on twist angle [40,41]. Achieving sub-degree rotational precision is essential for reproducibility but remains challenging in practice. Another major hurdle for 2D magnets in device applications is their low operating temperature (below 100 K), and attempts to raise it through ionic gating, chemical doping, or substrate proximity simply introduce new layers of unpredictability [34]. Additionally, unencapsulated 2D magnets suffer from rapid environmental degradation via oxidation and moisture absorption. Although encapsulation with hexagonal boron nitride (hBN) mitigates this issue, the capping layer’s thickness and interfacial quality inherently perturb the magnetic state via dielectric screening and chemical potential shifts. All these constraints define the key materials and fabrication challenges that limit the implementation of 2D magnetic materials for sensing.

Several recent review articles have summarized the rapid progress in 2D magnetic materials, with emphasis on fundamental magnetic properties, material synthesis, and spintronic device concepts (e.g., References [25,31,42]). Other works have examined magnetic sensing technologies within specific frameworks, such as magnetoelectric composites [43] or magnetic nanomaterials for biosensing [44], often encompassing material systems beyond just 2D materials. While these studies provide valuable insight into individual material platforms or sensing modalities, a unified and mechanism-oriented perspective on the use of 2D magnetic materials across diverse sensing applications remains less developed. This review addresses this gap by systematically examining multiple sensing mechanisms within a single framework and evaluating their respective advantages, limitations, and application potential.

Although research and application efforts surrounding 2D magnetic materials have been heavily concentrated on spintronic applications, the technological potential of these materials extends far beyond traditional data storage and logic operations. Consequently, this review shifts focus to a set of sensing mechanisms that have been experimentally demonstrated in 2D magnetic materials in order to examine how these selected mechanisms enable sensing functionalities in 2D magnetic materials. By examining the underlying transduction mechanisms, we detail how the intricate physical and structural complexities of these systems can be navigated to engineer ultrasensitive, flexible, and multifunctional sensors. Ultimately, this review aims to provide a mechanism-oriented perspective connecting fundamental 2D magnetism with the development of emerging sensing architectures.

The remainder of this review is organized as follows. Section 2 discusses tunneling magnetoresistance and its implementation in 2D magnetic systems. Section 3 examines magnetoelectric effects, followed by spin–orbit torque-based sensing in Section 4. Section 5 introduces antiferromagnetic resonance, while Section 6, Section 7, Section 8, Section 9 and Section 10 cover additional sensing mechanisms including field-dependent permeability, magneto-photoresponse, magnon-assisted photoconduction, spin-torque diode effects, and magnetostriction. Finally, Section 11 provides concluding remarks and an outlook on future research directions. These diverse sensing mechanisms span a wide range of transduction pathways, frequency regimes, and device architectures, and their relative advantages and limitations are synthesized and compared throughout this review to provide a comparative perspective on these emerging sensing approaches.

## 2. Tunneling Magnetoresistance

### 2.1. Mechanism

Tunneling magnetoresistance (TMR) is a quantum transport effect most commonly associated with magnetic tunnel junctions (MTJs), which serve as essential building blocks in modern spintronic technologies [45]. In a typical MTJ, two ferromagnetic electrodes are separated by an ultrathin insulating or semiconducting barrier that permits electron tunneling [45]. In this structure, the junction resistance is governed by the relative orientation of the magnetizations of the two ferromagnetic electrodes: the electrical resistance is lower when their magnetizations are aligned (parallel) and higher when they are opposed (antiparallel) [46].

There are two types of MTJs: spin-valve MTJs (sv-MTJ) and spin-filter MTJs (sf-MTJ) [45]. In a sv-MTJ, the two ferromagnetic electrodes are separated by a thin, non-magnetic insulator. Under parallel alignment, electrons from each spin sub-band in the first electrode can tunnel efficiently into the corresponding spin sub-bands of the second electrode, producing a low-resistance state. However, when the magnetizations are antiparallel, electrons encounter a mismatch between available spin states across the barrier, increasing electron scattering and thus creating a higher resistance. The TMR ratio quantifies this contrast between the antiparallel and parallel resistance states. In contrast, a sf-MTJ features a magnetic semiconducting or insulating layer sandwiched between non-magnetic electrodes. In such systems, spin-up and spin-down electrons experience different barrier energies, generating strongly spin-polarized currents. High TMR ratios can be achieved in sf-MTJs by either aligning multiple spin filter layers or using A-type antiferromagnets in combination with an external magnetic field [45].

TMR-based devices have become central to emerging memory and logic technologies [45,47]. Because the different resistance states of an MTJ are stable without continuous power, these devices provide an inherently non-volatile means of storing information, forming the basis of magnetic random-access memory (MRAM) and related spin-logic concepts [45,47]. Moreover, since magnetic fields can induce changes in the magnetic configuration of these devices, ultimately leading to measurable changes in electrical resistance, MTJs can be incorporated into devices to sense magnetic fields. For example, Nakano et al. [48] fabricated a TMR magnetic field sensor array using quasi-2D materials in combination with magnetic-flux concentrators, achieving sub-pT detectivity.

Recent advances in van der Waals heterostructures have enabled new approaches for realizing spin-dependent tunneling. In contrast to conventional thin-film (quasi-2D) MTJs that often exhibit rough interfaces and diminished performance at reduced dimensions, 2D magnetic materials provide atomically flat surfaces and highly uniform barrier thicknesses [45]. These features minimize interfacial disorder and support coherent spin-dependent tunneling, resulting in improved nanoscale device performance. The following section highlights recent developments in MTJs that utilize 2D magnetic materials. Readers seeking a more expansive examination of MTJs based on 2D materials are encouraged to consult the comprehensive review by Zhao et al. [45].

### 2.2. Demonstrations

**Figure 1:**
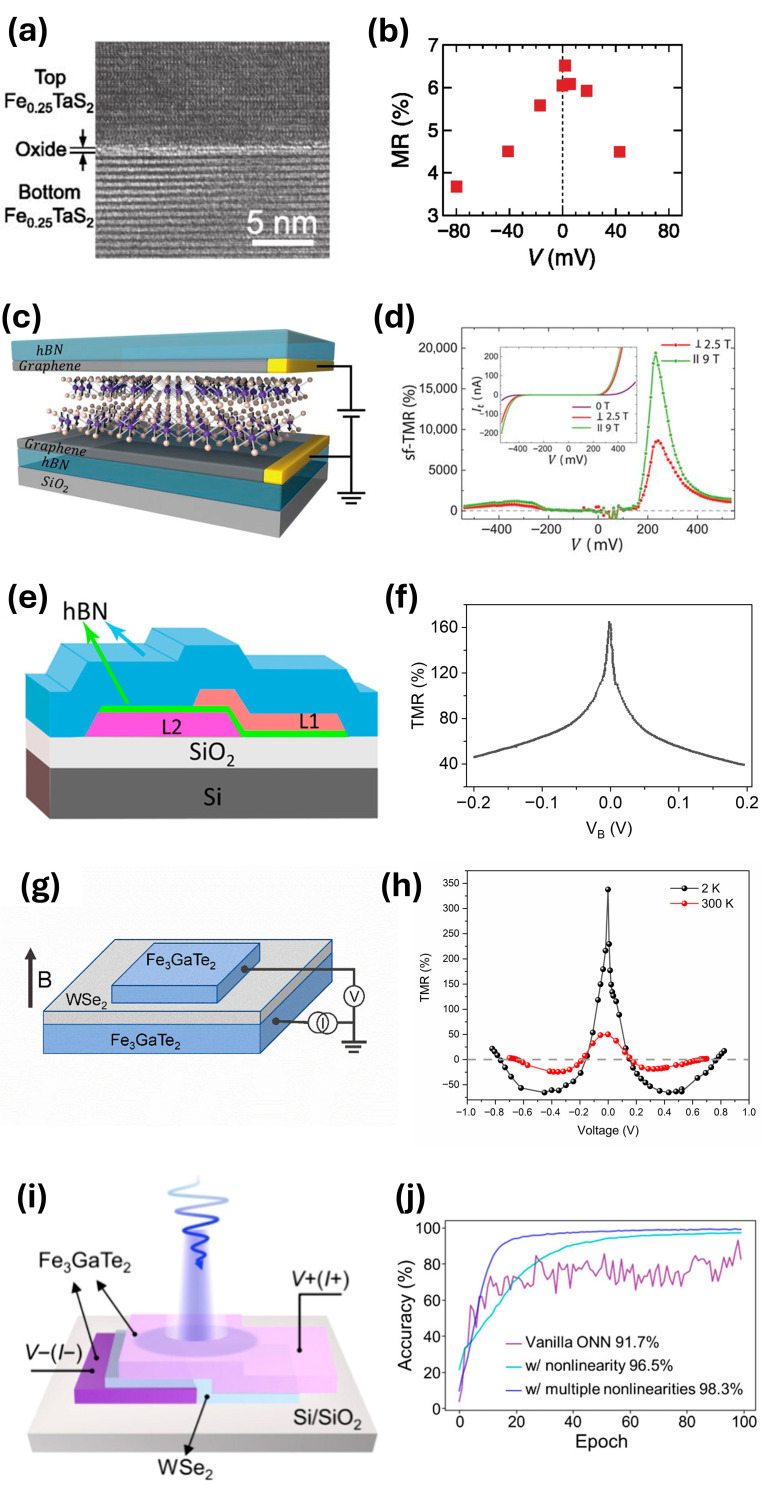
Each row shows a pair of figures from different MTJ studies. The left column shows the MTJ structure, while the right column shows performance characteristics. (**a**,**b**) Measurements taken at 5 K. Reproduced with permission from Arai et al. [49]. Copyright © 2015 AIP Publishing. (**c**,**d**) Measurements taken at 2 K. Reproduced with permission from Song et al. [17]. Copyright © 2018 The American Association for the Advancement of Science. (**d**) sf-TMR ratio was calculated from the data shown in the inset. (**e**,**f**) Measurements taken at 4.2 K. Reproduced with permission from Wang et al. [50]. Copyright © 2018 American Chemical Society. (**f**) Data was plotted by the authors of this work after digitizing the original TMR plot shown in Wang et al. [50]. (**g**,**h**) Measurements taken at 2 K and 300 K. Reproduced with permission from Pan et al. [51]. Licensed under the Creative Commons CC BY license. (**i**,**j**) The plot demonstrates how Zhu et al.’s optical neural network achieves better image recognition accuracy by leveraging the nonlinear photoresponse of their light-sensitive MTJs. Reproduced with permission from Zhu et al. [52]. Copyright © 2024 American Chemical Society.

Arai et al. [49] demonstrate a sv-MTJ device based on a ferromagnetic vdW heterostructure composed of two flakes of Fe_0.25_TaS_2_ separated by a thin oxide layer of Ta_2_O_5_ (Figure 1a). In this system, Fe-intercalated TaS_2_ (Fe_0.25_TaS_2_) acts as the ferromagnetic component and exhibits strong perpendicular magnetic anisotropy (PMA), making it suitable for spin-dependent tunneling structures. The device is fabricated by stacking the flakes after a native Ta_2_O_5_ oxide layer naturally forms on the surface, which serves as the tunnel barrier between the magnetic layers. This configuration enables the observation of TMR, with the reported TMR signal ratio reaching approximately 6% (Figure 1b). The authors note that higher TMR ratios could potentially be achieved through improved fabrication approaches, such as van der Waals transfer performed in an inert atmosphere to better preserve interface quality. In addition, device optimization was constrained by the size of available exfoliated flakes; flakes with thicknesses of only tens of nanometers were too small for device fabrication, requiring the use of thicker flakes of roughly 100 nm. A further limitation of the system is the relatively low Curie temperature of about 160 K, which restricts operation to below room temperature and presents a challenge for practical sensing applications.

Song et al. [17] demonstrate a sf-MTJ based on the vdW magnet CrI_3_. In this device, atomically thin CrI_3_ serves as the magnetic tunnel barrier between few-layer graphene electrodes (Figure 1c). The TMR originates from magnetic-field-driven transitions between layered antiferromagnetic and ferromagnetic configurations in CrI_3_. Transport measurements show discrete current plateaus corresponding to these magnetic states, which were confirmed using magneto-optical characterization.

The magnitude of the TMR increases strongly with CrI_3_ thickness, reaching approximately 310–530% in bilayer devices, 2000–3200% in trilayers, and up to ~19,000% in four-layer spin-filter junctions at low temperature (Figure 1d)—among the largest TMR values reported for magnetic multilayer structures. While these results demonstrate the potential for extremely high signal contrast, such performance is typically achieved under controlled experimental conditions and may not directly translate to practical device implementations. Additionally, the authors note that device behavior is sensitive to the environment and could potentially be tuned through improved fabrication conditions or electrostatic doping of the graphene contacts. However, a key limitation is that the devices operate only at low temperatures due to the intrinsic properties of CrI_3_.

Wang et al. [50] demonstrate spin valve behavior in vdW heterostructures composed of exfoliated Fe_3_GeTe_2_ electrodes separated by an hBN tunnel barrier, with an additional hBN capping layer to prevent oxidation (Figure 1e). The devices function as a sv-MTJ below the Curie temperature of ~220 K. Representative devices exhibit TMR of ~160% at 4.2 K (Figure 1f), with resistance changing from ~19 kΩ to 50 kΩ near magnetic transitions. Limitations include low-temperature operation, multilayer rather than atomically thin electrodes, and bias-dependent TMR suppression likely due to inelastic tunneling or spin relaxation. The authors suggest that future improvements could explore thinner exfoliated layers and electrostatic gating to further tune device performance.

Pan et al. [51] demonstrate a vdW MTJ based on Fe_3_GaTe_2_ electrodes separated by a few-layer WSe_2_ tunnel barrier and encapsulated with hBN (Figure 1g). The device exhibits spin-valve TMR that is electrically tunable at room temperature, allowing the readout of magnetic states with low operating currents. Representative measurements show TMR of ~340% at 2 K and ~50% at 300 K (Figure 1h), with operation maintained up to ~340 K. This capability enables bias-programmable control of the contrast between the resistances associated with the parallel and antiparallel magnetization states, providing an electrically adjustable magnetic-state readout.

Limitations include the reduction in TMR with increasing temperature and high-bias currents, as well as the need to maintain clean, symmetric interfaces to maximize performance. Nonetheless, the robust TMR observed near room temperature, combined with the Curie temperature of Fe_3_GaTe_2_, highlights the potential of such vdW heterostructures for compact, low-current magnetic sensors with in situ tunable offset, dynamic range, and transfer characteristics.

Zhu et al. [52] demonstrate a similar MTJ using a vdW heterostructure with Fe_3_GaTe_2_ electrodes separated by a 3.6 nm-thick p-type WSe_2_ tunnel barrier and encapsulated with hBN to prevent oxidation (Figure 1i). The ultrathin WSe_2_ layer provides high crystal quality and a wider band gap, enabling strong optical response, while the Fe_3_GaTe_2_ electrodes maintain a high Curie temperature of ~380 K. The junction exhibits magnetoresistance that depends on both the magnetization direction and the intensity of incident light, allowing the device to toggle between “on” and “off” states under a low bias of 0.1 V. Under 450 nm illumination, the photocurrent increases by three orders of magnitude compared to the dark state, with a response time below 100 ms, demonstrating rapid and robust light control of the MTJ.

When integrated into an optical neural network, this device enhances image recognition accuracy, reaching up to 98.3% (Figure 1j), by combining three functionalities in a single junction: light sensing through photocurrent generation, memory storage via magnetoresistance, and computing enabled by the nonlinear photoresponse coupled with magnetoresistance modulation. Limitations include operation at relatively low light intensities (~337 mW/cm^2^), which could be improved by using higher-absorptivity 2D semiconductors, reducing the thickness of ferromagnetic layers, or refining fabrication to reduce photocurrent noise. Overall, this work highlights the potential of light-sensitive MTJs for artificial intelligence (AI) hardware, providing a platform for in-memory sensing and computing that could advance MRAM-based machine vision systems.

Wang et al. [53] investigate TMR in vdW heterostructures composed of CrCl_3_ barriers sandwiched between graphite electrodes and encapsulated with hBN. The CrCl_3_ layers favor spin orientations that lie in-plane due to its small magnetic anisotropy, and the junction’s resistance serves as an electrical readout of the magnetic state. The device reveals both spin-flip and spin-flop transitions, with the latter occurring only in odd-numbered CrCl_3_ layers, highlighting an even–odd effect. Sharp jumps in the differential resistance mark the saturation (spin-flip) field, while minima at finite fields indicate spin-flop boundaries, allowing precise mapping of magnetic phase evolution with field and temperature.

The MTJ demonstrates magnetoconductance amplitudes of several hundred percent, with saturation occurring at critical fields ranging from ~1.1 T in bilayers to ~1.9 T in thicker CrCl_3_ stacks and Néel temperatures around 17–18 K. These measurements also enable extraction of interlayer exchange and layer magnetization parameters. Limitations include operation at cryogenic temperatures and weaker magnetoresponse compared to CrI_3_-based junctions, as well as sensitivity of small-field features to bias conditions. Nonetheless, this work establishes TMR as a compact probe of magnetic state in atomically thin antiferromagnets, offering a path to detect phase boundaries and state evolution, particularly if higher-temperature materials or engineered heterostructures are employed.

Across these studies, TMR-based sensors demonstrate high signal contrast and sensitivity relative to other approaches discussed in this review. Device performance varies significantly depending on junction characteristics and fabrication quality, which directly impact tunneling behavior and signal contrast. However, most reported devices remain constrained by low operating temperatures and variability arising from fabrication sensitivity. As a result, achieving robust, room-temperature operation while maintaining high TMR ratios and reproducibility remains a central challenge for translating these systems into practical sensing technologies.

## 3. Magnetoelectric Effects

### 3.1. Mechanism

Multiferroic materials are a class of material that exhibit two or more ferroic orders, such as ferroelectricity, ferromagnetism, antiferromagnetism, or ferroelasticity [54]. In such materials, it is possible for these ferroic orders to strongly couple and produce novel effects. If a multiferroic material is both ferroelectric and ferromagnetic and has sufficiently strong coupling, it may exhibit a magnetoelectric (ME) effect in which either an electric polarization is modulated (or a voltage is induced) by an external magnetic field, or a magnetization is induced by an external electric field. This interplay opens magnetoelectric materials to several applications ranging from sensors and oscillators to memory devices and filters [54].

While intrinsic, single-phase ME materials exist, they typically have Curie temperatures well below room temperature and don’t achieve high inherent ME couplings [54]. Achieving this coupling of ferroelectric polarization with magnetization within a single-phase material is difficult because ferroelectricity typically requires empty d orbitals, whereas magnetism favors partially filled d orbitals [55]. As such, effort has been made to induce ME coupling extrinsically by synthesizing ME composites. In a ME composite, neither material exhibits a ME effect on its own; rather, the two materials interact to generate an ME effect. The coupling between the ferroelectric and ferromagnetic materials in a ME composite is mediated by strain—specifically, the ferromagnetic material exhibits magnetostriction while the ferroelectric material exhibits piezoelectricity.

### 3.2. Demonstrations

He et al. [55] demonstrate a ME sensor that detects magnetic fields through the magnetocapacitive effect, in which the capacitance of a material changes as a function of an applied magnetic field. To overcome the limitations with single-phase ME materials, the authors employ a ferromagnetic/ferroelectric nanocrystal composite that enables magnetoelectric coupling through the interaction of two distinct material phases.

**Figure 2:**
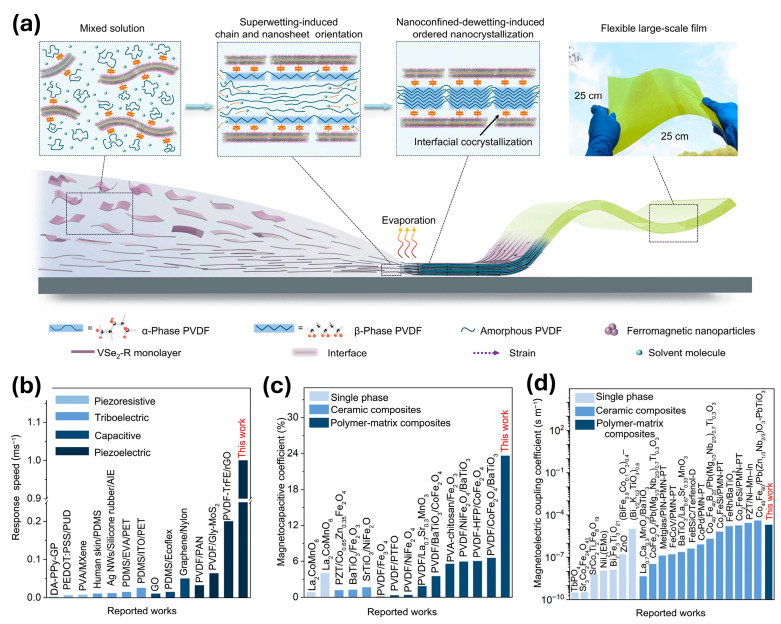
(**a**) Schematic of the fabrication process for the PVDF/VSe_2_-COOH composite film. (**b**) Response speed, (**c**) magnetocapacitance coefficient, and (**d**) magnetoelectric coupling coefficient compared to other magnetoelectric materials. Reproduced with permission from He et al. [55]. Copyright © 2025 The American Association for the Advancement of Science.

The device consists of a PVDF/VSe_2_-COOH composite film synthesized via interfacial cocrystallization (Figure 2a). The ferroelectric component is β-phase poly(vinylidene fluoride) (PVDF), while the ferromagnetic component is 2D VSe_2_ material functionalized with para-carboxybenzene groups (denoted VSe_2_-COOH). These functional groups enable lattice relaxation of the VSe_2_ monolayers within the polymer matrix, allowing a flat interface to form onto which PVDF crystallizes into its polar β phase without disrupting the intrinsic room-temperature ferromagnetism of VSe_2_. This engineered interface enables strong coupling between the ferromagnetic and ferroelectric components of the composite.

As a result, the sensor exhibits a magnetocapacitive coefficient of 23.6% (Figure 2c), a piezoelectric coefficient of 101.4 pm/V, and a piezoelectric voltage coefficient of 1180.6 × 10^−3^ Vm/N. The device also demonstrates an ultrafast response speed of approximately 1 ms^−1^ (Figure 2b), roughly an order of magnitude faster than conventional magnetic sensors. In addition to its high sensitivity, the composite film is flexible and energy-efficient, and its ME coupling allows detection of magnetic fields originating from multiple sources. These characteristics reflect the effectiveness of strain-mediated coupling in enabling magnetic field detection within composite architectures.

This demonstration highlights the potential of magnetoelectric coupling as a low-power and sensitive transduction mechanism for magnetic sensing. Compared to conventional Hall-effect and magnetoresistive sensors, such devices offer the advantages of room-temperature operation, mechanical flexibility, and potentially lower power consumption and fabrication cost. In particular, composite magnetoelectric architectures provide a pathway toward highly miniaturized and energy-efficient sensing platforms. Compared to tunneling magnetoresistance-based approaches discussed in Section 2, magnetoelectric sensors rely on strain-mediated coupling rather than spin-dependent electronic transport, providing an alternative transduction mechanism that can enable magnetic sensing in device architectures and operating regimes where transport-based approaches may be less practical. At the same time, performance depends on the effectiveness of coupling within the composite structure, which governs the efficiency of magnetic-to-electrical signal conversion. Further progress will depend on enhancing the strength of the magnetoelectric response to improve overall device sensitivity.

## 4. Spin–Orbit Torque

### 4.1. Mechanism

Spin–orbit torque (SOT) is the torque exerted on the magnetization of a ferromagnet as a current passes through a ferromagnet–heavy metal heterostructure [56]. In such systems, strong spin–orbit coupling converts the charge current into transverse spin currents, which in turn exerts a torque on the ferromagnetic layer. Two effects can emerge simultaneously that cause this torque: the bulk spin Hall effect and the interfacial Rashba–Edelstein effect due to broken inversion symmetry at the interface, each of which typically causes damping-like SOT and field-like SOT, respectively [57]. Precisely how much each of these effects contribute to the overall SOT is an open question [57] and depends on the materials and the interface characteristics [56]. Regardless, this interaction is capable of inducing measurable effects that can be exploited for sensing applications.

### 4.2. Demonstrations

Xie et al. [56] demonstrate a spin-torque gate sensor capable of detecting the angle of an external magnetic field by exploiting damping-like SOT in a ferromagnet/Weyl semimetal heterostructure. Damping-like SOT switching requires an external magnetic field component parallel to the current direction. By leveraging this requirement, the device can determine the orientation of an applied magnetic field based on the conditions under which magnetization switching occurs.

**Figure 3:**
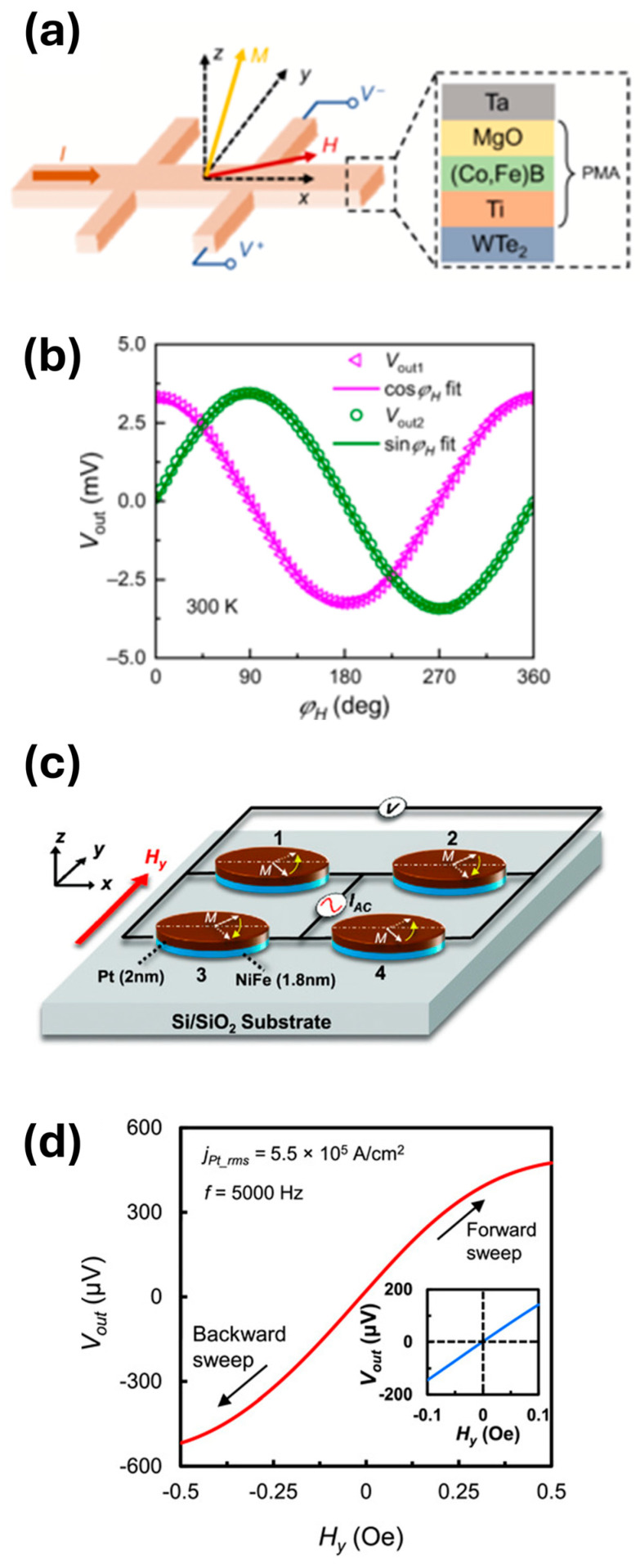
(**a**,**b**) Reproduced with permission from Xie et al. [56]. Copyright © 2021 American Physical Society. (**a**) Schematic of sensor device. (**b**) Voltage output as a function of magnetic field angle. (**c**,**d**) Reproduced with permission from Xu et al. [58]. Copyright © 2018 WILEY-VCH Verlag GmbH & Co. KGaA, Weinheim. (**c**) Schematic of Wheatstone bridge sensor. (**d**) Voltage output as a function of a sweeping magnetic field. Inset is also voltage output, but for a smaller sweeping range.

To realize this concept, the authors fabricate a multilayer Ta/MgO/(Co,Fe)B/Ti/WTe_2_ heterostructure (Figure 3a). The WTe_2_ layer acts as the primary SOT generator due to its strong spin–orbit coupling and large spin Hall effect, injecting spin current into the adjacent ferromagnetic layer. The (Co,Fe)B layer serves as the ferromagnetic element and forms an interface with MgO, which produces strong interfacial perpendicular magnetic anisotropy and improves thermal stability. Although (Co,Fe)B is a conventional three-dimensional magnetic material, the ultrathin film used in the device behaves as a quasi-2D magnetic layer. A 2-nm Ti layer functions as a seed layer that promotes perpendicular magnetic anisotropy while maintaining high spin transparency, thereby preserving efficient spin–orbit torque transfer. The Ta layer serves as a protective capping layer.

Using this architecture, the device operates as a magnetic field angle sensor by monitoring the switching behavior of the ferromagnetic layer under current excitation (Figure 3b). The sensitivity of the spin-torque gate is comparable to a spin Hall magnetoresistance (SMR) sensor previously demonstrated by the same research group, while its dynamic range is significantly larger, highlighting the advantages of the SOT-based sensing approach.

Xu et al. [58] demonstrate a magnetoresistive sensor that exploits spin Hall magnetoresistance (SMR) and SOT in an ultrathin ferromagnet/heavy-metal bilayer. In such systems, charge currents in the heavy metal generate spin currents via the spin Hall effect, which interact with the magnetization of the adjacent ferromagnetic layer. This coupling enables magnetic-field detection through changes in resistance while simultaneously allowing SOT to dynamically influence the magnetic state. Although the materials used are not strictly 2D, the nanometer-scale NiFe/Pt layers form a quasi-2D heterostructure, making the concept relevant to sensing platforms based on true 2D magnetic materials.

To exploit these effects, the authors fabricate a Wheatstone bridge composed of four ellipsoidal NiFe (1.8 nm)/Pt (2 nm) bilayer elements optimized for SMR and SOT operation (Figure 3c). The sensor is driven by an alternating current (AC), while its response to an external magnetic field is detected as a rectified direct-current (DC) voltage (Figure 3d). This configuration integrates AC excitation, domain stabilization, rectified readout, and DC offset cancellation directly within the ultrathin bilayer, eliminating the need for additional structures or circuitry typically used for sensor linearization and magnetic domain control.

The resulting device supports several sensing modes, including magnetic field detection, rotation-angle measurement, vibration sensing, and finger-motion tracking. Field sensing occurs when the AC-driven bridge produces a DC voltage proportional to the applied field, while angle detection arises from changes in output voltage as the field orientation varies. The device can also detect yaw vibrations at 1.16 Hz, exhibiting a higher signal-to-noise ratio than a commercial gyroscope despite a smaller signal amplitude, and can track finger bending through changes in the projection of Earth’s magnetic field along the sensor axis.

Performance measurements show near-zero DC offset, negligible hysteresis, and temperature-robust SMR, with a detectivity of approximately 1 nT/√Hz at 1 Hz and a sensitivity of about 0.17 mV/V/Oe, comparable to commercial anisotropic magnetoresistance sensors despite the ultrathin sensing layer. The demonstrations were conducted within metal shielding to remove Earth-field bias, and the sensor’s dynamic range is determined largely by the shape anisotropy of the patterned elements. Improved low-noise filtering, optimized AC excitation to eliminate residual offsets, and refined vibration-measurement setups could further enhance performance. These results highlight the role of device geometry, excitation conditions, and measurement configuration in determining the sensitivity and stability of SOT-based magnetic sensors.

These studies demonstrate that spin–orbit torque-based devices provide a flexible platform for magnetic sensing within the types of device architectures discussed here, combining high sensitivity with compatibility with established spintronic architectures. In particular, SOT-based device structures enable compact implementations with integrated excitation, stabilization, and signal detection. In contrast to magnetoelectric sensors (Section 3), which rely on coupled ferroic responses, SOT-based devices utilize current-driven magnetic excitation and electrical detection within the same device framework, enabling sensing through integrated excitation and readout. This level of functional integration offers a promising pathway toward scalable and miniaturized sensor technologies based on 2D magnetic materials. At the same time, device performance depends on the efficiency of spin-torque-driven signal generation and the effectiveness of signal extraction under applied excitation. Further progress will require improving signal extraction and reducing measurement noise to enable reliable detection under applied excitation.

## 5. Antiferromagnetic Resonance

### 5.1. Mechanism

When a ferromagnet is placed in an external magnetic field, its net magnetization undergoes precession about the field direction. This phenomenon, known as ferromagnetic resonance (FMR), typically occurs at microwave frequencies in the GHz range, enabling ferromagnets to function as both GHz-band sources and detectors.

In contrast, antiferromagnets contain multiple spin sublattices whose magnetic moments cancel in equilibrium. The strong exchange coupling between these sublattices produces an effective exchange field on the order of 10^2^–10^3^ T, which sets the characteristic antiferromagnetic resonance (AFMR) frequencies in the THz regime [59]. As a result, AFMR offers a pathway toward compact THz-frequency sources and detectors—capabilities that remain technologically limited.

### 5.2. Demonstrations

Cham et al. [60] demonstrate electrical detection and control of AFMR in a vdW heterostructure using spin-filter tunneling and spin-torque antiferromagnetic resonance (ST-AFMR). Although the work does not present a conventional sensing device that responds to an external stimulus, it establishes a platform for detecting and manipulating internal magnetic dynamics, which is relevant for future high-frequency sensing and signal-processing applications. Microwave currents excite AFMR, producing oscillations in the tunnel junction resistance. Mixing between the oscillating resistance and current generates a measurable DC voltage, enabling direct electrical readout of the resonance.

**Figure 4:**
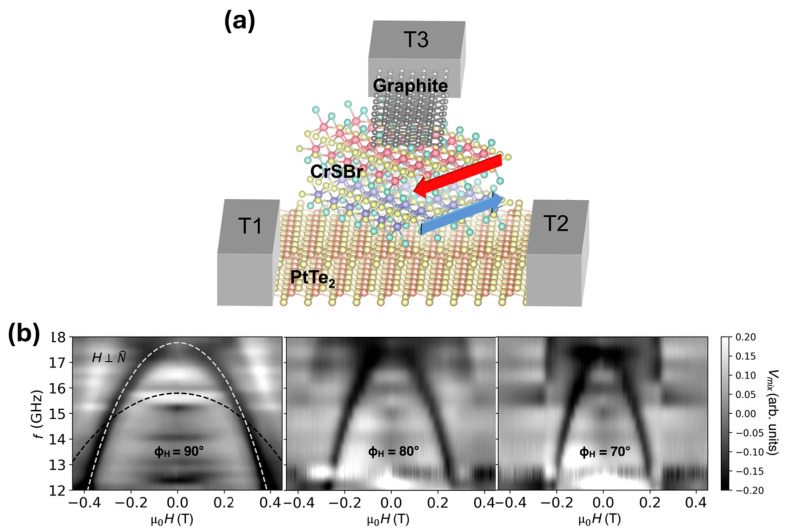
(**a**) Schematic of the heterostructure device. The red and blue arrows indicate the direction of the antiferromagnetic sublattices. (**b**) Spin-torque AFMR spectra for magnetic fields applied along three different angles. $\phi =90^{\circ }$ corresponds to the intermediate anisotropy axis. The dashed gray line represents the fitted field dependence of the optical AFMR mode, whereas the dashed black line shows the calculated field dependence of the undetected acoustic mode. Reproduced with permission from Cham et al. [60]. Copyright © 2025 The American Association for the Advancement of Science.

The device is a micron-scale three-terminal junction based on a vdW heterostructure composed of PtTe_2_/bilayer CrSBr/graphite, encapsulated with hBN (Figure 4a). The CrSBr layer acts as a spin-filter tunneling barrier and the antiferromagnetic material, with a Néel temperature of 132 K. The PtTe_2_ electrode, a vdW type-II Dirac semimetal, generates spin–orbit torque (SOT), which electrically tunes the resonance by modifying the damping of the antiferromagnetic dynamics.

This architecture allows selective detection of the optical AFMR mode, with resonance frequencies observed in the 12–16 GHz range (Figure 4b). Analysis of the field-dependent spectra enables extraction of key magnetic parameters, including the anisotropy and exchange fields (μ_0_H_a_ ≈ 0.34 T, μ_0_H_E_ ≈ 0.096 T, μ_0_H_c_ ≈ 0.77 T). The device achieves up to ~12% reduction in damping at current densities of 10^10^ A/m^2^ before heating effects become limiting. The three-terminal junction impedance (~700 Ω) is favorable for GHz operation compared with traditional graphite-based junctions.

Current limitations include low operating temperature, heating that limits damping reduction, and readout sensitivity confined to the optical AFMR mode. The SOT primarily acts on the CrSBr sublattice adjacent to PtTe_2_, which allows selective control but also constrains the device’s ability to achieve negative effective damping. Future improvements could include reducing PtTe_2_ thickness to minimize heating, optimizing the ratio of antiferromagnetic parameters, lowering intrinsic damping, patterning the junction into a nanowire oscillator, and applying SOT to both interfaces to approach negative effective damping.

This demonstration illustrates the potential of AFMR-based platforms for compact electrical detection and generation of high-frequency signals in the GHz to THz regime. Compared to tunneling magnetoresistance-based approaches (Section 2) and magnetoelectric sensors (Section 3), which primarily operate in lower-frequency regimes, AFMR-based devices provide access to much higher-frequency magnetic dynamics. By leveraging layered magnetic structures, these devices enable access to ultrafast spin dynamics without the presence of stray fields, making them promising for high-frequency sensing, modulation, and signal-generation technologies. However, practical implementation remains limited by the relatively narrow range of demonstrated operating frequencies, which currently fall within the GHz regime and remain below the ideal THz frequencies associated with antiferromagnetic dynamics. Further progress will depend on shifting the accessible frequency range toward the THz regime while maintaining efficient electrical detection of AFMR signals and enabling stable operation under ambient conditions.

## 6. Field-Dependent Permeability

**Figure 5:**
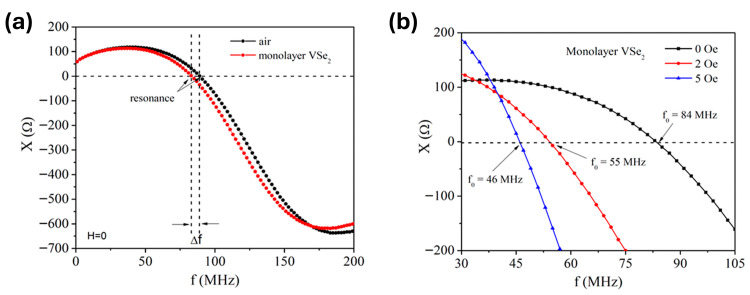
Sensor reactance as a function of frequency under (**a**) no external magnetic field for both air and monolayer VSe_2_ cores and (**b**) under different magnetic fields. Reproduced with permission from Jimenez et al. [61]. Licensed under the Creative Commons CC BY license.

Jimenez et al. [61] demonstrate a magnetic field sensor based on an LC resonator incorporating a 2D ferromagnetic core. The device consists of a Co_69.25_Fe_4.25_Si_13_B_12.5_Nb microwire coil wrapped around a monolayer VSe_2_ ferromagnetic layer grown on either MoS_2_ or highly oriented pyrolytic graphite (HOPG) substrates. In this configuration, the magnetic permeability of the VSe_2_ core varies with an applied magnetic field, modifying the inductive properties of the coil and producing a measurable shift in the LC circuit’s resonance frequency (Figure 5a,b). Unlike conventional induction coil sensors, which typically measure changes in inductance directly, this approach detects magnetic fields by monitoring the field-dependent resonant response of the LC circuit.

Using this architecture, the sensor achieves a sensitivity of approximately 1.6 × 10^7^ Hz/Oe, comparable to sensors based on bulk materials with ultrahigh magnetic permeabilities while maintaining minimal core losses. Importantly, the use of a 2D monolayer magnetic core enables significant miniaturization. Conventional induction coil sensors typically face a tradeoff between sensitivity and device size: increasing the number of coil turns or enlarging the coil improves sensitivity but increases the overall sensor dimensions. In contrast, incorporating a field-dependent ferromagnetic core allows the LC resonator to maintain high sensitivity even at small scales by exploiting changes in magnetic permeability.

Field-dependent permeability provides a relatively simple and direct mechanism for magnetic sensing, leveraging changes in inductive or impedance response. By integrating an atomically thin ferromagnetic core within a resonant detection architecture, such devices deliver high-sensitivity sensing in a reduced footprint, advancing compact implementations of 2D magnetic sensors. Compared to more complex spintronic approaches, this method provides straightforward readout schemes and potential compatibility with conventional circuit architectures. Unlike AFMR-based approaches discussed in Section 5, which target GHz to THz frequency regimes, permeability-based sensors rely on changes in inductive response and do not require high-frequency excitation, making them well suited for implementations that utilize simpler excitation and readout schemes. However, the sensing response relies on permeability-based modulation within a resonant detection scheme, which may limit flexibility in tuning the device response. Further improvements will depend on expanding the tunability of the sensing response while preserving the compact and efficient detection approach.

## 7. Magneto-Photoresponse

**Figure 6:**
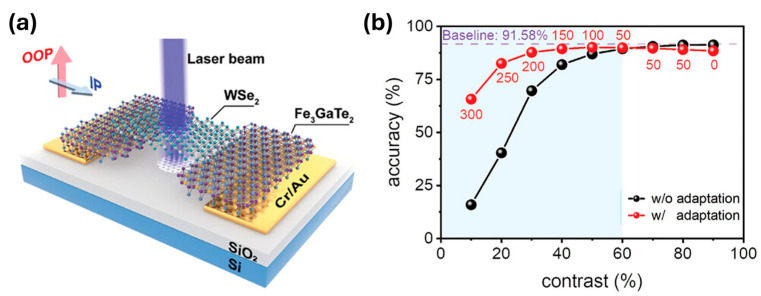
(**a**) Schematic of the photodetector. (**b**) Image-recognition accuracy as a function of contrast of the convolutional neural network with and without the magneto-photoresponse adaptation layer. The blue region shows the contrast regime in which adaptation leads to improved performance. Reproduced with permission from Zhu et al. [62]. Copyright © 2024 Wiley-VCH GmbH.

Zhu et al. [62] demonstrate a photodetector that exploits magneto-photoresponse, in which the photocurrent of a semiconductor is modulated by the magnetic state of an adjacent ferromagnet. In their design, the magnetic field effectively replaces the electrostatic gate typically used in phototransistors, enabling dynamic control of the device photosensitivity. The detector is constructed from a lateral van der Waals heterostructure composed of Fe_3_GaTe_2_/p-type WSe_2_/Fe_3_GaTe_2_ (Figure 6a), where the ferromagnetic Fe_3_GaTe_2_ electrodes modulate carrier transport in the WSe_2_ channel, enabling magnetic-field control of the photoconductive response. Through this configuration, the magnetic state of the Fe_3_GaTe_2_ layers influences carrier transport in the WSe_2_ semiconductor, allowing the device to function as a magnetically controlled, gateless phototransistor. This magnetic modulation is further leveraged to implement an in-sensor visual adaptation mechanism, which automatically adjusts the device response to improve the image contrast factor without external processing.

To evaluate sensing performance, the authors integrate the detector into a machine-vision framework using a ResNet20 convolutional neural network trained on the CIFAR-10 dataset, where image-recognition accuracy serves as a metric for photosensitivity. These results are compared against the same neural network operating without the magnetically controlled adaptation layer, which serves as the baseline (Figure 6b). Incorporating the magneto-photoresponse adaptation layer significantly improves recognition performance for low-contrast images. For example, recognition accuracy for 10% contrast images increases by approximately fourfold under a 300 mT magnetic field, while 20% contrast images show about a twofold improvement under 250 mT.

Several advantages arise from this architecture. The device exhibits a room-temperature magneto-photoresponse, whereas many magnetically modulated optoelectronic effects are limited to cryogenic operation. The gate-free design simplifies device structure and reduces power consumption, while the in-sensor visual adaptation capability eliminates the need for external processing circuitry, reducing latency and enabling more compact hardware designs suitable for high-density photodetector arrays with fewer electrical interconnects. Further performance improvements may be possible by replacing the WSe_2_ channel with narrower-bandgap 2D semiconductors, such as black phosphorus or graphene, which could enhance optical absorption and carrier generation.

This demonstration highlights magneto-photoresponse as a mechanism for coupling magnetic and optical degrees of freedom, enabling multifunctional sensing capabilities. By combining magnetic detection with optical readout, such devices provide a pathway toward integrated sensing and processing platforms, including machine-vision and adaptive optoelectronic systems. In contrast to the predominantly electrically and magnetically coupled sensing mechanisms discussed in earlier sections, this approach introduces direct coupling between magnetic and optical signals, introducing additional sensing modalities compared to earlier approaches discussed in this review. At the same time, device performance depends on material selection and heterostructure design, which can pose challenges for scaling to larger-area implementations. Continued progress in material synthesis and device integration will be critical for realizing the full potential of magneto-optoelectronic sensing platforms.

## 8. Magnon-Assisted Photoconduction

**Figure 7:**
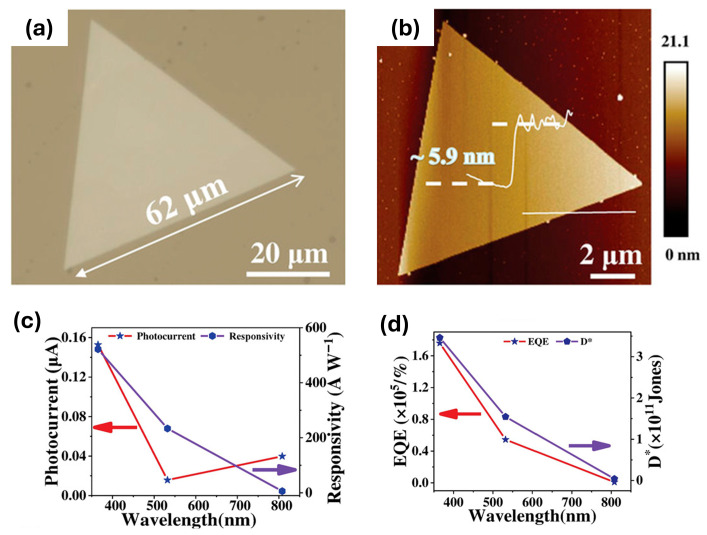
(**a**) Optical image of an α-MnSe crystal. (**b**) AFM image of an α-MnSe crystal. The inset shows a height profile across the white horizontal line in the image. (**c**) Photocurrent and (**d**) external quantum efficiency (EQE) and specific detectivity (D*) as a function of illumination wavelength. Reproduced with permission from Zhou et al. [63]. Licensed under the Creative Commons CC BY license.

Zhou et al. [63] report a photodetector based on an ultrathin, quasi-2D film of the antiferromagnetic semiconductor α-MnSe (Figure 7a,b), in which the optical response arises from both conventional interband absorption and spin-sensitive, magnon-mediated sub-bandgap transitions. The device supports broadband detection across 365–808 nm. The efficiency of photocarrier generation is influenced by the antiferromagnetic spin configuration, with these spin-dependent processes most pronounced below the Néel temperature of ~160 K. Additional sub-bandgap absorption arises from defect states in the film, further extending the spectral response.

Under 365 nm illumination, the detector exhibits high responsivity (521.8 A/W), detectivity (3.46 × 10^11^ Jones), and an external quantum efficiency of 1.76 × 10^5^% (Figure 7c,d). While these values demonstrate the potential for high photoresponse, they are obtained under controlled experimental conditions and may not directly reflect performance in practical device environments. Temperature-dependent measurements reveal that photocurrent increases with temperature, whereas responsivity decreases, reflecting the interplay between thermal activation, spin ordering, and defect-mediated absorption. Cooling the device to 80 K reduces the influence of trap states and enhances the alignment of spin order, resulting in faster response times (rise: 0.29 s vs. 3.5 s at 300 K; decay: 0.56 s vs. 6.8 s at 300 K) and improved photocurrent linearity with incident power compared to room temperature operation.

The ultrathin character of α-MnSe enables efficient light–matter interaction while maintaining spin-dependent effects, but performance is currently limited by low carrier mobility (~0.88 cm^2^/V·s) and trap-state density, which constrain switching speed, photocarrier transport, and overall device efficiency. Additionally, response time is slower than photodetectors based on other materials such as CdTe or 2D RhI_3_. Future improvements could arise from enhanced film quality, optimized contacts and interfaces, and reduced trap-state density, which would increase carrier mobility and improve response speed. Overall, this work illustrates how magnon-assisted processes in 2D antiferromagnets can be harnessed for broadband, spin-sensitive photodetection, offering a platform for exploring magnetically active optoelectronic devices.

This work demonstrates that magnon-assisted photoconduction provides a pathway for detecting magnetic excitations through optical means, linking spin dynamics and charge transport. In particular, magnon-assisted processes in 2D antiferromagnets enable spin-sensitive photodetection and suggest opportunities for magnetically active optoelectronic functionalities. However, the approach remains at an early stage of development, with challenges related to limited carrier transport, slow response times, and the need for low-temperature operation below the Néel temperature. Further progress will require improving carrier transport and response speed while enabling operation under more practical conditions to support efficient photodetection applications.

## 9. Spin-Torque Diode Effect

**Figure 8:**
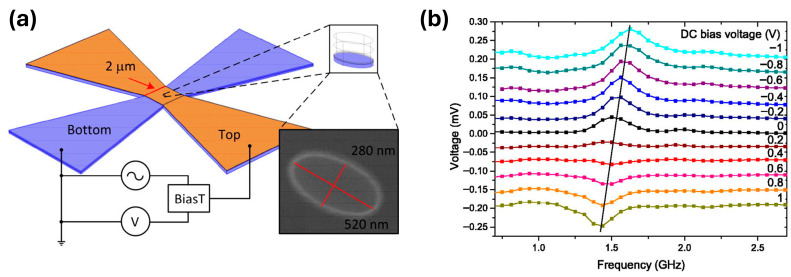
(**a**) Schematic of the MTJ, including the RF generator and voltmeter. The elliptical nanopillar at the center is the quasi-2D heterostructure. (**b**) Spin-torque ferromagnetic resonance signal using bias voltages ranging from −1 to 1 V with a perpendicular external magnetic field of 600 Oe. Reproduced with permission from Skowroński et al. [64]. Copyright © 2014 AIP Publishing.

Skowroński et al. [64] demonstrate a voltage-tunable radiofrequency (RF) detector based on a magnetic tunnel junction (Figure 8a), that exploits the spin-torque diode effect in combination with voltage-controlled magnetic anisotropy (VCMA). When an alternating current passes through the MTJ, the resulting spin-polarized current exerts spin-transfer torque on the free magnetic layer, driving magnetization precession near its ferromagnetic resonance frequency. The resulting resistance oscillations mix with the RF current flowing through the junction, producing a rectified DC voltage across the device that serves as the RF detection signal. The detector is implemented using quasi-2D magnetic materials to form a heterostructure of Pt_38_Mn_62_ (16 nm)/Co_70_Fe_30_ (2.1 nm)/Ru (0.9 nm)/Co_40_Fe_40_B_20_ (2.3 nm)/MgO (1.6 nm)/Co_40_Fe_40_B_20_ (1–2 nm).

Analysis of the ferromagnetic resonance response shows a Lorentzian lineshape, indicating that the rectified voltage signal originates primarily from spin-transfer torque rather than from VCMA or perpendicular torque contributions. Voltage-controlled magnetic anisotropy nevertheless provides a means of tuning the resonance conditions of the device. By varying the bias voltage across the MTJ, the magnetic anisotropy of the free layer is modified, which shifts the frequency at which the magnetization precession occurs. As a result, the RF detection band can be adjusted between approximately 1.4 and 1.6 GHz, corresponding to a tuning rate of about −97 MHz/V (Figure 8b). Measurements performed over a bias range of approximately ±1 V also allow the device to be used to characterize spin-transfer torque components through analysis of the mixing voltage.

Despite its tunability, the present device exhibits several performance limitations. The quality factor is relatively low, and the junction shows strong impedance mismatch with the RF circuitry, resulting in 99.5% of the incident RF power being reflected. In addition, the comparatively high resistance of the MTJ restricts bandwidth to about 2.5 GHz. Potential improvements include increasing the junction area to reduce resistance and improve impedance matching, as well as employing point-contact or vortex-based structures to increase the quality factor and enhance detection sensitivity.

This demonstration illustrates that spin-torque diode-based sensing offers a route for detecting high-frequency signals through rectification of spin dynamics, combining sensitivity with compact device design. Such systems are particularly attractive for microwave detection and signal processing applications, where their frequency tunability can be leveraged. Nevertheless, the performance of these devices remains influenced by the limitations discussed above, which affect the achievable detection performance in current implementations. Further progress will depend on addressing these factors to enable reliable and effective high-frequency sensing in practical applications.

## 10. Magnetostriction

Jiang et al. [65] demonstrate a nanoelectromechanical system (NEMS) resonator that uses magnetostriction in a 2D magnetic material to transduce magnetic and mechanical stimuli into shifts in mechanical resonance frequency. Magnetostriction occurs when changes in magnetization produce lattice strain, which can modify the mechanical properties of a structure. In this platform, magnetic-field-induced changes in the magnetization of CrI_3_ generate strain within the resonator membrane, altering the device’s resonance frequency. This coupling between magnetism, strain, and mechanical motion creates an intrinsic transduction mechanism that converts magnetic or mechanical stimuli directly into measurable shifts in resonant frequency.

**Figure 9:**
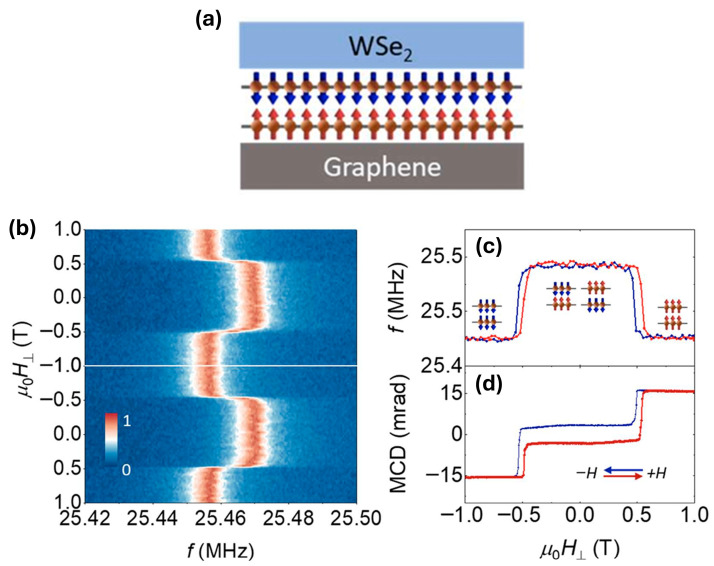
(**a**) Resonator membrane schematic composed of bilayer antiferromagnetic CrI_3_ sandwiched between graphene and monolayer WSe_2_. The arrows show the spins in the CrI_3_ layers. (**b**) Normalized vibration amplitude as a function of driving frequency and perpendicular magnetic field. (**c**) Resonance frequency and (**d**) magnetic circular dichroism measurements of a bilayer CrI_3_ resonator as a function of external magnetic field. The arrows in (**c**) indicate the spin orientations in the bilayer CrI_3_. (**c**,**d**) The red curves indicate negative-to-positive sweeping of the magnetic field, while the blue curves indicate positive-to-negative sweeping. Reproduced with permission from Jiang et al. [65].

The resonator consists of a suspended graphene/CrI_3_/WSe_2_ heterostructure clamped across microtrenches to form drumhead-type NEMS membranes (Figure 9a). The CrI_3_ layer serves as the magnetic element, while graphene encapsulation protects the air-sensitive material and helps maintain structural integrity. The WSe_2_ layer provides additional environmental protection and also functions as a strain gauge; the exciton resonance energy of monolayer WSe_2_ redshifts linearly with strain at a rate of approximately 63 meV/% strain. Devices incorporating both bilayer and six-layer CrI_3_ were investigated. Mechanical motion of the suspended membrane is detected using optical interferometry to measure out-of-plane displacement. The extremely low mass of the atomically thin membrane enables very small strain changes to produce large shifts in resonance frequency, and the resonator exhibits a relatively high quality factor of approximately 2500, enabling precise frequency-based readout.

Magnetic field sensing arises from the strong coupling between magnetization and mechanical strain in the CrI_3_ layer. When an out-of-plane magnetic field is applied, the system undergoes spin-flip transitions that produce abrupt changes in the mechanical resonance frequency. These discrete transitions enable threshold-type magnetic field detection, with bilayer devices exhibiting a single transition near ~0.5 T (Figure 9b–d) and six-layer devices showing two transitions at ~0.9 T and ~1.8 T. In contrast, in-plane magnetic fields cant the spins rather than inducing discrete transitions, resulting in smooth and monotonic frequency shifts. This behavior enables analog magnetic field sensing, allowing the field strength to be inferred from the resonance frequency up to approximately 6 T, where the frequency shift saturates.

The coupling between strain and magnetization also enables inverse magnetostrictive effects. Mechanical strain applied to the resonator shifts the magnetic spin-flip field by roughly 32 mT, which in turn modifies the resonance frequency. Although the authors do not explicitly frame it as a sensing modality, the demonstrated inverse magnetostrictive effect in which strain shifts the spin flip field by ~32 mT implies that the platform could, in principle, transduce mechanical stimuli into measurable magnetic or mechanical resonance changes. More broadly, the platform demonstrates that mechanical resonators based on 2Dmagnets can provide a means of detecting magnetic phases and phase transitions through purely mechanical measurements. The same magnetostrictive coupling could also enable magnetically driven actuation, where magnetic fields induce motion of the membrane.

Several challenges remain for practical implementation. Experiments were performed at cryogenic temperatures (~4 K), and the air sensitivity of CrI_3_ necessitates encapsulation with protective layers such as graphene and WSe_2_. In addition, fabrication processes involving exfoliation, transfer, and trench formation can introduce contamination or mechanical imperfections such as residues or wrinkles in the suspended membranes. Despite these limitations, the work highlights the potential of magnetostrictive NEMS platforms for non-electrical probing of magnetic phases, spin textures, and phase transitions in 2D vdW magnets, complementing conventional transport and magneto-optical characterization methods.

This demonstration illustrates the potential of magnetostrictive nanoelectromechanical systems for sensing applications by coupling magnetic and mechanical degrees of freedom, enabling detection through frequency shifts and mechanical resonance. These devices offer high sensitivity and the possibility of integrating multiple sensing modalities within a single platform. However, their operation often requires low temperatures and complex fabrication processes, which currently limit scalability and practical deployment. Further progress will depend on enabling operation under more practical conditions while maintaining sensitivity and functionality, as well as developing fabrication approaches that support reliable and scalable device implementation.

## 11. Conclusions

Two-dimensional magnetic materials represent an emerging platform for next-generation sensing technologies. Their atomic thickness, strong electrostatic tunability, and compatibility with van der Waals heterostructures provide capabilities that are difficult to achieve using conventional bulk magnetic materials. As discussed throughout this review, these attributes enable a diverse set of sensing mechanisms, including those discussed in this review: tunneling magnetoresistance, magnetoelectric coupling, spin–orbit torque, antiferromagnetic resonance, field-dependent permeability, magneto-photoresponse, magnon-assisted photoconduction, spin-torque diode detection, and magnetostrictive nanoelectromechanical systems. Collectively, these approaches demonstrate that 2D magnets can function as active sensing elements across multiple sensing domains, including magnetic fields, mechanical strain, optical signals, and high-frequency electromagnetic radiation. The studies highlighted throughout this review represent demonstrations of emerging sensing concepts and illustrate the capabilities of 2D magnetic materials, although further work is needed to assess their scalability and reproducibility across different material systems and device architectures.

While these demonstrations highlight the potential of 2D magnetic sensing across the mechanisms discussed here, the relative advantages, limitations, and technological readiness of the different approaches vary significantly. Across the sensing mechanisms discussed, clear trade-offs emerge between sensitivity, operating conditions, and device complexity. These trade-offs reflect not only engineering challenges but also intrinsic differences in the underlying sensing mechanisms, which fundamentally shape their performance and application space. Tunneling magnetoresistance-based devices exhibit some of the highest signal contrast and are among the most mature architectures, but often require low-temperature operation in 2D systems. Achieving robust room-temperature operation remains a key priority, which may require the development of air-stable 2D ferromagnets or the use of proximity-induced magnetism in engineered heterostructures. Magnetoelectric sensors offer low-power operation and room-temperature functionality, though their performance depends strongly on composite engineering. Advancing these systems toward practical applications will require fabrication strategies that enable uniform and efficient strain transfer across large-area composite structures. Spin–orbit torque and spin-Hall-based devices provide integrated functionality and scalability, but require careful materials optimization to achieve high sensitivity. Optical and optoelectronic approaches, including magneto-photoresponse and magnon-assisted photoconduction, enable multifunctional sensing and in-sensor processing, though they remain relatively early-stage and may face challenges related to speed and material quality. Progress in this area will depend on improving response speed and ensuring stable operation under ambient conditions while maintaining sensitivity. Meanwhile, emerging concepts such as magnetostrictive NEMS and antiferromagnetic resonance offer unique transduction mechanisms and access to new frequency regimes, but are currently limited by operating conditions and fabrication complexity.

In terms of future potential, approaches that combine room-temperature operation, scalability, and system-level integration appear particularly promising. TMR-based vdW heterostructures remain strong candidates for near-term applications due to their high sensitivity and compatibility with existing spintronic technologies, especially as materials with higher Curie temperatures are developed. Magnetoelectric composites also present a compelling pathway for low-power and flexible sensing platforms. Additionally, magneto-optoelectronic and multifunctional heterostructure devices are emerging as a powerful direction for integrated sensing and computing, particularly in machine-vision and neuromorphic systems. In contrast, mechanisms such as antiferromagnetic resonance and magnetostrictive NEMS, while scientifically rich, are likely to remain longer-term opportunities due to current limitations in operating temperature and device complexity. From an application perspective, these sensing mechanisms are naturally suited to different operational regimes, with tunneling magnetoresistance and magnetoelectric approaches aligning with high-sensitivity and low-power sensing, respectively, while high-frequency techniques such as antiferromagnetic resonance and spin-torque-based detection offer opportunities in GHz to THz signal processing and communication technologies. Overall, the most viable pathways toward practical 2D magnetic sensors will likely be those that balance high sensitivity with room-temperature operation and scalable fabrication, while also leveraging, where appropriate, the unique material integration capabilities of van der Waals materials.

A key advantage of 2D magnetic materials lies in their ability to integrate seamlessly into multifunctional heterostructures. By stacking magnetic layers with semiconducting, insulating, or optically active materials, device architectures can combine sensing, signal processing, and memory functions within a single nanoscale platform. Examples discussed in this review include magnetic tunnel junctions with extremely large tunneling magnetoresistance ratios, magnetoelectric composites capable of rapid low-power magnetic detection, and magnetically tunable optoelectronic devices that perform adaptive image processing directly within the sensor. These demonstrations illustrate how the intrinsic properties of 2D magnets can be leveraged to create compact, energy-efficient sensing platforms with capabilities extending beyond those of traditional magnetic sensors.

Despite this progress, several challenges must be addressed before widespread practical implementation becomes feasible. Many currently studied 2D magnets exhibit magnetic ordering temperatures well below room temperature, limiting device operation to cryogenic environments. Environmental stability also remains a significant concern, as many layered magnets are sensitive to oxidation and moisture, necessitating encapsulation strategies that can influence device performance. In addition, scalable synthesis and reproducible device fabrication remain difficult due to the reliance on exfoliated flakes and the sensitivity of magnetic properties to layer thickness, stacking configuration, and interfacial quality.

Ongoing advances in materials discovery, heterostructure engineering, and device fabrication are expected to progressively overcome these limitations. Efforts aimed at identifying air-stable, high-Curie-temperature 2D magnets, improving wafer-scale growth techniques, and developing robust encapsulation strategies will be particularly important for translating laboratory demonstrations into practical technologies. At the same time, continued exploration of novel transduction mechanisms—especially those that couple magnetism with optical, mechanical, and quantum degrees of freedom—may further expand the range of sensing modalities accessible with 2D materials.

Looking forward, the application landscape for two-dimensional magnetic materials is expected to expand significantly as key materials and device challenges are addressed. In the near term, applications in miniaturized and high-sensitivity magnetic field sensing, including biomedical instrumentation and wearable electronics, represent particularly promising directions due to the ability of atomically thin materials to enable close-proximity detection and enhanced signal-to-noise ratios. Flexible and low-power sensing platforms may further enable continuous monitoring and human–machine interfacing. In parallel, the integration of magnetic functionality with optical and electronic degrees of freedom is opening pathways toward multifunctional devices capable of simultaneous sensing, memory, and signal processing, with demonstrated potential in machine-vision and in-sensor computing architectures. In this context, the choice of sensing mechanism will likely be guided by the required balance between sensitivity, operating conditions, and device complexity, with different approaches offering distinct advantages depending on the target application. At higher frequencies, concepts based on antiferromagnetic resonance and spin-torque effects provide opportunities for compact GHz and THz detectors and signal-processing technologies, although these remain longer-term prospects. Bridging the gap between these demonstrations and real-world systems will require continued advances in material stability, interface engineering, and scalable fabrication for large-area integration. Furthermore, realizing these applications relies on developing device architectures capable of reproducible and reliable operation under ambient conditions. 

Overall, the rapid progress achieved over the past decade suggests that two-dimensional magnetic materials are expected to play an increasingly important role in future sensor technologies. By combining extreme miniaturization with rich tunability and heterostructure compatibility, these materials provide a versatile platform for the development of ultrasensitive, multifunctional sensing systems suited for applications spanning wearable electronics and biomedical instrumentation to high-frequency communication and intelligent machine-vision hardware.

## Data Availability

No new data was created for this work.

## Abbreviations

The following abbreviations are used in this manuscript:

2D	Two-dimensional
AC	Alternative current
AFMR	Antiferromagnetic resonance
AI	Artificial intelligence
DC	Direct current
FMR	Ferromagnetic resonance
hBN	Hexagonal boron nitride
HOPG	Highly oriented pyrolytic graphite
ME	Magnetoelectric
MRAM	Magnetic random-access memory
MTJ	Magnetic tunnel junction
NEMS	Nanoelectromechanical system
PMA	Perpendicular magnetic anisotropy
PVDF	Poly(vinylidene fluoride)
RF	Radiofrequency
RKKY	Ruderman–Kittel–Kasuya–Yosida
sf-MTJ	Spin-filter magnetic tunnel junction
SMR	Spin Hall magnetoresistance
SOT	Spin–orbit torque
ST-AFMR	Spin-torque antiferromagnetic resonance
sv-MTJ	Spin-valve magnetic tunnel junction
TMR	Tunneling magnetoresistance
VCMA	Voltage-controlled magnetic anisotropy
vdW	van der Waals
